# Population‐Based Study of Incidence of Acute Abdominal Aortic Aneurysms With Projected Impact of Screening Strategy

**DOI:** 10.1161/JAHA.115.001992

**Published:** 2015-10-20

In the article by Howard et al, “Population‐Based Study of Incidence of Acute Abdominal Aortic Aneurysms With Projected Impact of Screening Strategy,” which published online August 19, 2015, and appeared in the August 2015 issue of the journal (*J Am Heart Assoc*. 2015;4:e001926 doi: 10.1161/JAHA.115.001926), the copyright statement has been amended to indicate a CC‐BY Open Access license.

The online version of the article has been updated and is available at http://jaha.ahajournals.org/content/4/8/e001926.

